# Reliability and validity of the Polish version of the Core Outcome Measures Index for the neck

**DOI:** 10.1007/s00586-013-3129-2

**Published:** 2013-12-23

**Authors:** Grzegorz Miekisiak, Mariusz Banach, Grzegorz Kiwic, Lukasz Kubaszewski, Jacek Kaczmarczyk, Adam Sulewski, Wojciech Kloc, Witold Libionka, Dariusz Latka, Marta Kollataj, Rafal Zaluski

**Affiliations:** 1 Department of Neurosurgery, Specialist Medical Center, Polanica-Zdroj, Poland; 2 Department of Neurosurgery, St. Raphael’s Hospital, Kraków, Poland; 3 Department of Neurosurgery, Regional Specialist Hospital, Jastrzebie-Zdroj, Poland; 4 Department of Orthopedics, University of Medical Sciences, Poznan, Poland; 5 Department of Neurology and Neurosurgery, Faculty of Medical Sciences, University of Varmia and Masuria, Olsztyn, Poland; 6 Department of Neurosurgery, Pomeranian Traumatology Center, Gdańsk, Poland; 7 Department of Neurosurgery, Regional Medical Center, Opole, Poland; 8 Department of Pain Research and Treatment of Jagiellonian University, Kraków, Poland; 9 Department of Neurosurgery, Medical University of Wroclaw, Wrocław, Poland

**Keywords:** Patient reported outcomes, COMI, Spine Tango, Validation, Neck pain

## Abstract

**Purpose:**

Patient reported outcome measures play an increasingly important role in the outcomes research. The Core Outcome Measures Index (COMI) is a short, multidimensional instrument initially developed for the use by patients with low back pain. This study is an evaluation of a Polish version of COMI adapted for neck pain.

**Methods:**

One hundred twenty-three patients complaining of neck pain were enrolled. All of them completed a questionnaire booklet containing COMI-neck, Neck Disability Index and Likert-type questions regarding the frequency of use of pain medications and pain frequency. Ninety-eight patients returned the retest questionnaire. Data quality was also assessed. Assessment of psychometric properties included examination of data quality, construct validity, test–retest reliability and factor analysis.

**Results:**

The quality of data was good with no missing answers and a little floor effect. Exploratory factor analysis revealed a single-factor structure. Reliability expressed as intraclass correlation coefficient was 0.88 (95 % CI 0.84–0.92) for the overall COMI score and was generally good for most of individual core items. The minimum detectable change (MDC_95%_) was 1.97.

**Conclusion:**

This version of the COMI-neck is a valid and reliable instrument, with good psychometric properties. It can be recommended for Polish-speaking patients.

**Electronic supplementary material:**

The online version of this article (doi:10.1007/s00586-013-3129-2) contains supplementary material, which is available to authorized users.

## Introduction

Neck pain is a very common complaint with a 12-month prevalence ranging between 30 and 50 % [1]. Despite widespread occurrence, understanding of the underlying mechanism remains poor and, as a result, even most popular treatment options are often challenged. Increasing pressure from patients, payers and governing agencies leads to an increased demand for unbiased measures for the outcome research. In case of subjective, hard to validate symptoms such as neck pain, reliable assessment is a difficult task. Therefore, a dedicated class of instruments has been introduced to facilitate collection of data from patients, known as patient reported outcome measures (PROMs).

PROMs play a pivotal role in the assessment of efficacy of treatment for various musculoskeletal disorders. They provide an invaluable insight into patients’ perception of their health and the effects of treatment in a scientific fashion. A well-designed PROM above all should excel in three psychometric aspects: reliability, validity and responsiveness. The validity of a measurement is an assessment of the extent to which it measures what it purports to measure. Reliability requires that an instrument is reproducible and internally consistent, while responsiveness in this context addresses whether an instrument is sensitive to changes of importance to patients [2]. It is also important to have a short and simple questionnaire, which reduces the response and processing burden to both patients and caregivers.

Several disease-specific PROMs have been proposed for the evaluation of neck pain and related complains such us Neck Disability Index (NDI) [3], NASS-cervical [4], Copenhagen Neck Functional Disability Scale [5] or Northwick Park Neck Pain Questionnaire [6]. Over the years, NDI has arguably become most popular [7]; however, none of the instruments can be considered as a golden standard. As questionnaires must cover all necessary questions, to provide adequate validity, they are often long and complicated [8]. This approach limits their clinical application in daily practice when efficiency is of paramount importance. Last but not the least, complexity can negatively affect the process of translation and cross-cultural adaptation [9].

Deyo et al. [10] recommended a set of six core questions, known as the Core Outcome Measures Index (COMI), which is actually a relatively short and valid instrument designed to assess outcome measures for patients with low back pain. The questions cover several dimensions such as pain (axial and radiating to the extremity), function, symptom-specific well-being, quality of life and disability (social and work). The evaluation of psychometric properties was encouraging [11]. COMI was accepted as a main PROM for the Spine Tango—the international spine surgery registry of Eurospine, the Spine Society of Europe (SSE) [12]. Thereafter, soon multilingual versions COMI were cross-culturally adapted [13–17]; this allows the use of COMI in international studies and registries. COMI was adapted for the cervical spine with some minor changes such as enquiring about neck rather than back problems. This version also showed good validity and reliability [18].

The objective of our study was to perform a validation of the COMI-neck questionnaire for Polish-speaking patients with detailed evaluation of psychometric properties.

## Materials and methods

### The Core Outcome Measures Index

The COMI is a self-administered multiple choice questionnaire containing seven items designed for quantitative evaluation of five domains (pain, difficulties in everyday life, symptom-specific well-being, general quality of life and the social and work disability). The first two items assess the axial and limb radiating pain (originally back and leg pain) with a visual analog scale ranging from 0 to 10. Following items are rated on 5-point Likert-type scales. The social and work disability questions refer to the last 4 weeks preceding evaluation, the rest pertains to the last 7 days. COMI score is calculated by averaging the values for each of five domains after re-scoring them in 0–10 scale. For the pain domain, the higher of the two values is used and for disability it is an arithmetic mean of social and work disability. The Polish version (PL) of the COMI-neck was derived from the previously translated and validated Polish COMI-back [16] with minor adjustment to address neck rather than back problems.

### Patients and the questionnaire booklet

A total of 123 patients from seven departments were enrolled, and received two questionnaire booklets [*n* = 123: 43 men, mean (SD) age 53.16 (7.55); 80 women mean (SD) age 49.93 (8.71)]. The inclusion criteria include neck pain lasting more than 4 weeks, pain with or without radiation to the arm/shoulder, age 18 years or above and good comprehension of the Polish language. Majority of patients (all but nine) were surgical candidates. Ninety-eight patients (79.67 %) returned the completed retest questionnaire within 2–14 days after the baseline test administration. There were no therapeutic interventions between administrations. Besides the COMI-neck questionnaire, the booklet also contained a previously validated Polish version of the NDI [7] and two Likert-type questions regarding the frequency of use of pain medications (“never” to “always”) and pain frequency (“never” to “always”). Included in the booklet was an information explaining the patients’ voluntary participation in the study. The study was approved by the local ethical committees.

### Statistical analyses

The COMI-neck score was calculated as described above. The NDI score was presented as percentage (maximum of 100). No missing items were allowed for COMI and Likert scales; no more than 20 % of missing data for the NDI was allowed. Floor and ceiling effect was determined by calculating the proportion of respondents who obtained highest (100) and lowest (0) possible COMI-neck scores in baseline questionnaires. For these subjects, no improvement or deterioration could be detected as they are already at the extremes. Thus, a high percentage of such responders would negatively affect the measures [13, 15]. Desired value for floor/ceiling effect is <15–20 % [19, 20], and values >70 % are considered detrimental [13, 15].

Construct validity refers to the degree to which the analyzed tool actually measures the construct being investigated. Convergent validity is a type of construct validity, and can be defined as the extent to which different measures that are designed to tap the same construct correlate with each other [21]. We assessed convergent validity by evaluating the correlation between the overall COMI-neck score and the NDI score. In addition, the relationship was tested between the COMI-neck score and two Likert-type questions (the frequency of use of pain medications and the frequency of pain) with scores treated as ordinal variables. Spearman rho (*σ*) corrected for ties was used in all correlation analyses. For the purpose of this study, the following thresholds for validity coefficients were accepted: *r* > 0.8 as excellent, 0.61–0.8 very good, 0.41–0.6 good, 0.21–0.4 fair, and 0–0.2 poor [22].

Exploratory factor analysis with principal components extraction was performed on all items to examine the latent dimensions of the scale. The optimum number of factors was determined by the number of eigenvalues >1. Item loadings on each factor ≥0.4 were considered satisfactory for inclusion in that factor [23].

Test–retest reliability is a measure of instruments’ consistency and stability over time. It is evaluated by repeated application of the test. In our study, the time interval between the questionnaire administrations was 2–14 days with no therapeutic interventions within this period.

The intraclass correlation coefficient (ICC) at the 95 % confidence interval (CI) was used for evaluating this form of reliability. The ICC can fall within the range 0.00–1.00, values from 0.60 to 0.80 indicate good reliability and above 0.80 are considered excellent [24]. Standard error of measurements (SEM) was used to establish the absolute measurement error and to calculate the minimum detectable change at the 95 % confidence level (MDC_95%_) for the instrument [13, 25]. The MDC_95%_ indicates the minimum change of score which can be considered by the patient a “real change”, greater than the instruments’ measurement error. At the 95 % confidence level, this can be calculated with a formula 1.96 × √2 × SEM, equivalent to 2.77 × SEM [26].

## Results

### Score distribution and missing data

The overall COMI-neck score was normally distributed according to the Kolmogorov–Smirnov test, yet the same test failed to present normality per each individual item score. There were no missing data for the COMI-neck questionnaire; three patients missed one question each of the NDI sub form.

### Floor and ceiling effect

The results were not adversely affected by the floor and ceiling effect (Table 1), majority of items fell within the desired range of <20 %. The floor effect was more prominent, with the highest value for symptom-specific well-being (59.35 %).

**Table 1:** Floor and ceiling effects

Core items (scoring)	Mean (SD)	Ceiling effect (best health) (%)	Floor effect (worst heath) (%)
Pain (0–10)	6.26 (2.73)	5.69	8.94
Function (1–5)	3.61 (0.86)	3.25	8.13
Symptom-specific well-being (1–5)	4.31 (1.05)	3.25	59.35
Quality of life (1–5)	3.55 (0.85)	2.44	10.57
Social disability (1–5)	3.3 (1.28)	8.94	23.58
Work disability (1–5)	2.9 (1.44)	20.33	21.14
Overall COMI score (0–10)	6.54 (2.04)	0.81	0.81

### Convergent validity

A very good correlation was observed between the COMI and NDI overall scores (Table 2). Each item of COMI correlated well with the NDI score. There was no statistically significant correlation between pain frequency and COMI. There was some negative but significant correlation between COMI and frequency of pain medication use (*σ* = −0.25, *p* < 0.05).

**Table 2:** Relationship between COMI, NDI and Likert-type questions expressed as Spearman’s rho

	NDI	Pain frequency	Frequency of pain medications
Pain (0–10)	0.63^†^	−0.05	−0.27^†^
Function (1–5)	0.61^†^	−0.01	−0.14
Symptom-specific well-being (1–5)	0.41^†^	0.00	−0.27^†^
Quality of life (1–5)	0.58^†^	0.06	−0.24^†^
Social disability (1–5)	0.49^†^	0.03	−0.19
Work disability (1–5)	0.50^†^	0.11	−0.22^†^
Overall COMI score	0.65^†^	0.04	−0.25^†^

^†^Statistically significant correlation with *p* < 0.05

### Exploratory factor analysis

A single factor was extracted with the exploratory factor analysis, which accounted for 61.6 % of the variation within the questionnaire. All items were highly loaded on this factor (Table 3).

**Table 3:** Factor loadings for single-factor solution of the Polish COMI-neck (component matrix)

Item	Factor 1
Function	0.868
Quality of life	0.829
Social disability	0.788
Pain	0.786
Work disability	0.722
Symptom-specific well-being	0.701

### Test–retest validity

The mean interval between questionnaire occasions was 8.17 days (SD 4.89, range 2–14). The score variations between applications were minor (Table 4). For all items, values were within ±10 % of agreement. The most consistent item was “pain” (82.65 % fell within ±10 % retest interval), the least was “work disability” (70.41 %), and for overall COMI score this value was 76.53 %.

**Table 4:** Test–retest validity

	Mean first (SD)	Mean retest (SD)	ICC (95 % CI)	SEM	MDC_95%_	MDC_95%_ in %
Neck pain (0–10)	5.29 (2.81)	5.30 (2.92)	0.736 (0.630–0.815)	1.44	4.00	39.99
Arm/shoulder pain (0–10)	5.06 (2.88)	5.34 (2.94)	0.828 (0.754–0.882)	1.19	3.31	33.09
Back function (1–5)	3.58 (0.94)	3.61 (0.85)	0.814 (0.734–0.871)	0.41	1.12	11.23
Symptom-specific well-being (1–5)	4.34 (0.96)	4.28 (1.11)	0.563 (0.411–0.684)	0.63	1.76	17.58
Quality of life (1–5)	3.44 (0.96)	3.52 (0.88)	0.797 (0.712–0.86)	0.43	1.2	11.98
Social disability (1–5)	3.23 (1.31)	3.27 (1.29)	0.839 (0.769–0.889)	0.53	1.46	14.56
Work disability (1–5)	2.79 (1.42)	2.89 (1.47)	0.889 (0.839–0.924)	0.47	1.31	13.1
Overall COMI score (0–10)	6.38 (2.04)	6.48 (2.07)	0.878 (0.824–0.917)	0.71	1.97	19.74

Acronyms explained in text

The reliability assessed by ICC was very good for most items, with the ICC (95 % CI) for overall COMI score 0.878 (0.839–0.924). The lowest ICC noted for the “symptom-specific well-being” item was 0.563 (0.411–0.684). The resulting SEM for the COMI score was 0.71, thus the minimum detectable change (MDC_95%_) was 1.97 (19.74 %).

## Discussion

PROMs are questionnaires designed to provide patients’ perception on health and the effects of treatment. Their role in contemporary healthcare is increasing. They have been used in numerous applications such as national audits [27], clinical trials [28], and surgical registries [29]. Well-developed PROMs are precision instruments that accurately assess patients’ health status [30] in specific domains. In order to improve the validity, the PROMs often tend to get lengthy and complicated, thus too burdensome for day-to-day application. The COMI was proposed as a short and robust alternative to prolonged symptom-specific questionnaires. Initially designed for the assessment of low back pain related disability, it was later also adopted for cervical spine [18] and for patients undergoing hip arthroplasty [31]. Its favorable psychometric properties have been proven by numerous published reports for both lumbar and cervical applications [18, 31, 32].

The PL COMI-neck is a slightly modified version of the previously validated and translated COMI-back [16]. This approach, rather than full translation and cross-cultural adaptation, was chosen to ascertain compatibility between these two versions. Besides, the original COMI-neck was a simple derivative of the lumbar version [18]. Although, one can assume that the PL COMI-neck should possess similar psychometric properties to the lumbar version, a dedicated validation was required for precise clinical applications, e.g., by providing the MDC_95%_.

The PL COMI-neck score was normally distributed, unlike the PL COMI-back [16] where the scores were positively skewed—it is possible, that in our present study, there were a substantial number of patients qualified for surgery for reasons other than pain (e.g., cervical spondylotic myelopathy). The results were not affected by either the floor or ceiling effects. The item, “symptom-specific well-being” had a significantly higher floor effect than other items, but it did not exceed 60 %. Values for majority of items were lower than 10 %. Our results are even better than data reported previously [18].

According to earlier reports [18, 33], either individual COMI-neck items or the overall COMI score showed a good correlation with a reference scale such as NDI and with the Spearman’s rho falling within the range of 0.41–0.65. The lowest value was noted for the item “symptom-specific well-being”. Similar observation was made by Fankhauser et al. [18]. There was no correlation between any of the items and the Likert-type question regarding the pain frequency. Interestingly, there was a small but significant correlation between the COMI scores and the frequency of pain medications used. It is possible that this is a matter of medication effectiveness in alleviating pain.

The exploratory factor analysis confirmed the robustness of COMI-neck and its mono-factorial structure. Previous studies on both COMI-back [14–16] and COMI-neck [33] scales, showed very good reliability for the test–retest analysis. The ICC for the overall COMI-neck score was 0.878, and the SEM and the MDC_95%_ were 0.71 and 1.97, respectively. These values are in agreement with previous reports for COMI-back [14–16] and COMI-neck [33]. The lowest value was for “symptom-specific well-being”.

## Conclusions

The PL COMI-neck is a valid and reliable instrument, and can be recommended for Polish-speaking patients. Its brevity compared with full-length questionnaires makes it an attractive option for everyday use, especially in busy environments, where reduction of data burden is essential. It can be integrated into the Polish module of the Spine Tango Registry or used in other international studies as the number of other language versions is constantly growing.

## Electronic supplementary material

Below is the link to the electronic supplementary material.

Supplementary material 1 (DOCX 17 kb)
