# Supplementary material for: Reliability and validity of the Polish version of the Core Outcome Measures Index for the neck
Source: Eur Spine J. 2013 Dec 23;23(4):898–903. doi: 10.1007/s00586-013-3129-2 (PMC3960435; doi:10.1007/s00586-013-3129-2)
Supplement: Supplementary file 1 — Supplementary material 1 (DOCX 17 kb) [file 586_2013_3129_MOESM1_ESM.docx]

Schorzenia odcinka szyjnego kręgosłupa mogą prowadzić do bólu karku i/lub bólu ręki/ramienia, a także do zaburzeń czucia takich jak mrowienia, kłucie czy drętwienie w każdej z wymienionych okolic.

1. Który z poniższych problemów jest dla Pana/Pani **najbardziej dokuczliwy**? Proszę zaznaczyć **JEDEN**

- ból karku
- ból ręki/ramienia
- zaburzenia czucia w karku/ręce/ramieniu np. mrowienie, kłucie, drętwienie
- żaden z powyższych

2. W następnych 2 pytaniach (2a i 2b) prosimy o zaznaczenie nasilenia bólu, poprzez wybranie odpowiedniej cyfry (gdzie „0”=brak bólu, „10”=najgorszy ból, jaki możesz sobie wyobrazić). Osobne pytania odnoszą się do **bólu karku** i **bólu promieniującego do ręki/ramienia.**

2a. Jak silny był **ból karku** w ostatnim tygodniu?

| brak bólu | 🞎 | 🞎 | 🞎 | 🞎 | 🞎 | 🞎 | 🞎 | 🞎 | 🞎 | 🞎 | 🞎 | najgorszy wyobrażalny ból |
| --- | --- | --- | --- | --- | --- | --- | --- | --- | --- | --- | --- | --- |
|  | 0 | 1 | 2 | 3 | 4 | 5 | 6 | 7 | 8 | 9 | 10 |  |

2b. Jak silny był **ból ręki/ramienia** w ostatnim tygodniu?

| brak bólu | 🞎 | 🞎 | 🞎 | 🞎 | 🞎 | 🞎 | 🞎 | 🞎 | 🞎 | 🞎 | 🞎 | najgorszy wyobrażalny ból |
| --- | --- | --- | --- | --- | --- | --- | --- | --- | --- | --- | --- | --- |
|  | 0 | 1 | 2 | 3 | 4 | 5 | 6 | 7 | 8 | 9 | 10 |  |

3. W ciągu **ostatniego tygodnia** jak bardzo schorzenie kręgosłupa **przeszkadzało Panu/Pani w normalnej pracy** (dotyczy zarówno pracy poza domem jak i prac domowych)?

- wcale
- nieznacznie
- średnio
- dosyć mocno
- ekstremalnie

4. Jeśli **resztę życia miał(a)by Pan/Pani spędzić z objawami, które odczuwa Pan/Pani obecnie**, jak by się Pan/Pani z tym czuł/a?

- bardzo zadowolony(a)
- dosyć zadowolony(a)
- ani zadowolony(a) ani niezadowolony(a)
- dosyć niezadowolony(a)
- bardzo niezadowolony (a)

5. Proszę zastanowić się nad **ostatnim tygodniem** swojego życia. Jak opisze Pan/Pani jakość swojego życia?

- bardzo dobra
- dobra
- średnia
- zła
- bardzo zła

6. **W ciągu ostatnich 4 tygodni**, przez ile dni musiał Pan/Pani **ograniczyć codzienne czynności** (praca, prace domowe, szkoła, sport i rekreacja) z powodu problemów z odcinkiem szyjnym kręgosłupa?

- wcale
- pomiędzy 1 a 7 dni
- pomiędzy 8 a 14 dni
- pomiędzy 15 a 21 dni
- więcej niż 22 dni

7.**W ciągu ostatnich 4 tygodni** przez ile dni dolegliwości ze strony kręgosłupa szyjnego **zmusiły Pana/Panią do pozostania w domu** (uniemożliwiły pójście do pracy, szkoły, wykonywanie zajęć domowych)?

- wcale
- pomiędzy 1 a 7 dni
- pomiędzy 8 a 14 dni
- pomiędzy 15 a 21 dni
- więcej niż 22 dni
